# An Account of an Anomalous Disease of the Tongue and Feet, Occurring in the Interior of Indiana

**Published:** 1845-02

**Authors:** Graham N. Fitch

**Affiliations:** Professor of Obstetrics, &c., in Rush Medical College; Logansport, Ind.


*An account of an Anomalous Disease of the Tongue and Feet, occurring in the interior of Indiana.* By GRAHAM N. FITCH, M. D., Professor of Obstetrics, &c., in Rush Medical College.

In calling the attention of the profession to a disease, no description of which has it ever been my fortune to meet, it may happen, that I shall be relating the symptoms and appearance of a pathological condition with which many are already familiar, either from practical observation, or through the medium of some one of the numerous periodical medical publications of the day, to which I have not access. Indeed, that many physicians in the interior of Indiana, where alone I have been cognizant of the disease, are practically familiar with it, I have the evidence afforded by mutual consultation; besides its prevalence at different periods, with such peculiarity and severity of symptoms, as necessarily to attract attention. The first cases prescribed for by me, came under notice in the spring of 1835; and scarce a year has elapsed since, without the presentation of more or less, although they have been, some years, more than ordinarily numerous. In the winter and spring of '35, '38, '41, and '44, its occurrence was more frequent than during the intermediate periods. It appears to be more prevalent among adults, paying no regard to sex. It commences with a burning sensation in the tongue, sometimes described as a stinging heat, not necessarily accompanied or preceded by any symptoms indicative of derangement of digestive or other organs. In truth, the reverse is usually the case, every function appearing to be healthily performed, until after the long continuance of the disease. This burning sensation increases, with varied rapidity, until it arrives to such a degree as to be productive of very considerable and constant distress, more or less increased upon taking anything hot or stimulating into the mouth. The tongue, at this stage, presents no unusual appearance, except perhaps an unnaturally *clean*, slightly florid appearance, at tip or edges. It appears, so far, to be a strictly local disease, and may now undergo a spontaneous cure, or yield to some domestic remedy; or partially disappear, perhaps entirely, to return after a varied interval, with increased severity. Whether thus temporarily checked or not, its return or continuance is soon marked by an aggravation of the burning pain. The tongue becomes bright red on the tips, edges, and along the centre, the space between the centre and edge, being often covered with a white loose crust. This, however, soon disappears in the progress of the disease, the entire tongue becoming of a deep red, resembling in color, raw flesh, but smooth, glossy, and moist. *It diminishes in every diameter*, length, breadth, and thickness, becoming remarkably sharpened at its apex. The papillæ all disappear, except a few of the larger at its base. A copious secretion of hot saliva adds to the patient's misery. The angles of the mouth and the lips externally, become, in some cases, excoriated or cracked. The sub-maxillary glands, even the cervical absorbent glands swell, but do not often present external evidences of inflammation—are not painful or very tender. Longitudinal cracks become perceptible in the tongue; sometimes one in the course of the *raphe*, and one immediately on each side. In other cases, there is but the one central crevice, but this often attains size sufficient to admit a small quill. The crevices have no appearance of having been produced by ulceration, but by a shrinking of the lateral muscular substance of the tongue. The pain becomes greatly increased, but retains its burning character. The appetite remains but little impaired, or if not, its loss is attributable more frequently to the local pain, than to any derangement of the stomach. There is an inability to take into the mouth any substance, solid or fluid, of any temperature, except perhaps one or two articles of the blandest kind. Some can comfortably consume a sufficient quantity of boiled milk and rice, or milk and soft bread. Others experience as much pain from efforts to eat these mild articles, as those of more stimulating nature. Nearly all, however, can chew fresh elm bark, and experience great comfort from holding it in their mouths. The pulse becomes accelerated, but not hard, rather irritable. There is some thirst. Except in those cases in which there is an inability to take any nutriment, and the naturally resulting emaciation and debility, the patient is able to attend to his or her ordinary avocations, until the supervention, in some cases, of another symptom, which will be soon mentioned. The longitudinal crevice in the tongue, as I before remarked, has not the appearance of an ulcer. No pus can ever be detected in it, or in any part of the mouth. I have never been able to discover apthæ or ulcerations, though that such do not exist in some cases, I will not assert, There is no unusual fœtor of the breath; the gums remain unaffected, as does indeed every part of the mouth, except the tongue and salivary glands.

After a continuance of these symptoms for an indefinite period, they gradually yield to medicinal treatment, or disappear spontaneously in the course of the summer. In many, (a majority,) the recovery appears to be complete. In others, and by no means a small proportion, a secondary affection, even more distressing than the primary one, manifests itself. As the lingual distress and appearances I have described, subside, "a burning sensation," as near like the one first felt in the tongue, as the difference in structure of the parts affected can be supposed to permit, becomes to be perceptible in the *bottoms of the feet.* The pain in this situation soon becomes intense, spreading from the soles to the superior parts of the feet, though never reaching the ancle joints. It deprives the patient of quiet by day, and rest by night; inducing him, for the purpose of relieving the intolerable sense of heat, to leave the feet at all times uncovered. Some have applied ice, or ice-cold water to the feet, for the same purpose; a proceeding usually productive of unpleasant consequences. There is not at first any tenderness. This however occurs in the further progress of the complaint, though seldom beyond a degree sufficient to produce some pain or flinching in walking. There *is at no time any*, or if any, very little, redness or swelling of the feet, and but little increase of temperature. Indeed, I have never been able to satisfy myself of the existence of any redness or swelling, other than what was produced by remedial applications.

This secondary affection is not so liable to follow more severe attacks of the original disease, (that of the tongue,) as the milder. Perhaps, when the tongue is long and severely affected, the disease expends itself in its original seat, thereby relieving the system from any disposition to attacks of it elsewhere; or the early occurrence in the feet of the symptoms above described, may act as a derivative, and consequently be soon followed by a subsideuce of of the pain in its first locality. I have known the peculiar (burning) pain to alternately attack the tongue and feet, disappearing from the one, almost simultaneously with its invasion of the other. It is rarely co-existent in both situations, and never in any severity. If the feet are once seized by it, they are generally the last part affected; as, if they are relieved by its recurrence in the tongue, (which does not often happen,) that relief is but temporary, the disease invariably returning to the feet, there to exhaust itself, or yield to treatment.

I have not, so far, succeeded in tracing it to any satisfactory cause. It has appeared in some instances, to be connected as an effect, with the consumption of an undue proportion of salted animal food. That such ever is the cause, it would be presumptuous to assert, with my limited means of observation. No opportunity for post-mortem examination has been offered me, never having witnessed a fatal termination.

Logansport, Ind., Jan. 1845.

[We are promised by Dr. Fitch, a continuation of the above article, describing the mode of treatment found most successful, and the details of several cases. We hope to present it to our readers in the next number.—Ed.]

